# Supplementary material for: Does surgical approach affect Hirschsprung-associated enterocolitis risk? A comparison between transanal Swenson-like and endorectal pull-throughs
Source: PLoS One. 2026 Jan 8;21(1):e0340813. doi: 10.1371/journal.pone.0340813 (PMC12782437; doi:10.1371/journal.pone.0340813)
Supplement: S2 Table — (DOCX) [file pone.0340813.s002.docx]

**Supplementary Table 2.** Post-hoc Chi-square Adjusted Residuals Analyses of Risk Factors for HAEC and non-HAEC across Swenson-like pull-through (TSLPT) and transanal endorectal pull-through (TEPT) groups.

| **Groups** | **Cut-off for HAEC score** | **Variable** | **Category** | **Outcome** | | **Interpretation vs expected frequency** |
| --- | --- | --- | --- | --- | --- | --- |
|  |  |  |  | **H (ASRESID)** | **nH (ASRESID)** |  |
| **TSLPT** | ≥4 | Sex | Female | +0.5 | –0.5 | No cells with ≥ 2 (no significant post-hoc findings) |
|  |  |  | Male | –0.5 | +0.5 |  |
|  |  | Age at Surgery | Neonate | –0.4 | +0.4 |  |
|  |  |  | Post-neonate | +0.4 | –0.4 |  |
|  |  | Nutritional Status | Normal | –0.7 | +0.7 |  |
|  |  |  | Underweight | +0.7 | –0.7 |  |
|  |  | Hemoglobin Level | Anemia | –0.7 | +0.7 |  |
|  |  |  | Normal | +0.7 | –0.7 |  |
|  |  | Albumin | Hypoalbuminemia | –1.1 | +1.1 |  |
|  |  |  | Normal | +1.1 | –1.1 |  |
| **TSLPT** | ≥10 | Sex | Female | –0.7 | +0.7 | No cells with ≥ 2 (no significant post-hoc findings) |
|  |  |  | Male | +0.7 | –0.7 |  |
|  |  | Age at Surgery | Neonate | –0.3 | +0.3 |  |
|  |  |  | Post-neonate | +0.3 | –0.3 |  |
|  |  | Nutritional Status | Normal | –0.5 | +0.5 |  |
|  |  |  | Underweight | +0.5 | –0.5 |  |
|  |  | Hemoglobin Level | Anemia | –0.5 | +0.5 |  |
|  |  |  | Normal | +0.5 | –0.5 |  |
|  |  | Albumin | Hypoalbuminemia | –0.7 | +0.7 |  |
|  |  |  | Normal | +0.7 | –0.7 |  |
| **TEPT** | ≥4 | Sex | Female | +0.5 | –0.5 | No cells with ≥ 2 (no significant post-hoc findings) |
|  |  |  | Male | –0.5 | +0.5 |  |
|  |  | Age at Surgery | Neonate | –0.7 | +0.7 |  |
|  |  |  | Post-neonate | +0.7 | –0.7 |  |
|  |  | Nutritional Status | Normal | –0.6 | +0.6 |  |
|  |  |  | Underweight | +0.6 | –0.6 |  |
|  |  | Hemoglobin Level | Anemia | –1.4 | +1.4 |  |
|  |  |  | Normal | +1.4 | –1.4 |  |
|  |  | Albumin | Hypoalbuminemia | ***–2.3*** | ***+2.3*** | HAEC is less frequent in hypoalbuminemic patients |
|  |  |  | Normal | ***+2.3*** | ***–2.3*** | HAEC is more frequent in patients with normal albumin level |
| **TEPT** | ≥10 | Sex | Female | +0.7 | –0.7 | No cells with ≥ 2 (no significant post-hoc findings) |
|  |  |  | Male | –0.7 | +0.7 |  |
|  |  | Age at Surgery | Neonate | - | - |  |
|  |  |  | Post-neonate | - | - |  |
|  |  | Nutritional Status | Normal | –0.5 | +0.5 |  |
|  |  |  | Underweight | +0.5 | –0.5 |  |
|  |  | Hemoglobin Level | Anemia | –0.5 | +0.5 |  |
|  |  |  | Normal | +0.5 | –0.5 |  |
|  |  | Albumin | Hypoalbuminemia | –0.7 | +0.7 |  |
|  |  |  | Normal | +0.7 | –0.7 |  |

*ASRESID = All adjusted residual values.

*Cells with a value of ASRESID ≥ 2 indicate significant deviation from expected frequencies.

*H = HAEC

*nH = non-HAEC
