# Supplementary material for: Does surgical approach affect Hirschsprung-associated enterocolitis risk? A comparison between transanal Swenson-like and endorectal pull-throughs
Source: PLoS One. 2026 Jan 8;21(1):e0340813. doi: 10.1371/journal.pone.0340813 (PMC12782437; doi:10.1371/journal.pone.0340813)
Supplement: Attachment — Submitted filename: Paper review Hirschsprung.docx [file pone.0340813.s003.docx]

**Paper review**

**Does surgical approach affect Hirschsprung-associated enterocolitis risk? A comparison between transanal Swenson-like and endorectal pull-throughs**

**General statement:**

The study by Azzahra et al, aimed to analyze the difference in the incidence of Hirschprug-associated enterocolitis following Transanal endorectal pull through (TEPT) compared to transanal Swenson-like pull-through (TSLPT). The application of Chi square test of independence/ Fisher’s exact test, and Multivariate analysis (logistic regression) to identify independent predictors of HAEC are appropriate for this study.

However, the following comments should be taken note of

**Comments/ Clarifications:**

**Abstract: This section needs to be re-written**

1) The statement, “Hirschsprung-associated enterocolitis (HAEC) is a complication of Hirschsprung disease (HSCR) that occurs before and after surgery.” May be changed to “Hirschsprung-associated enterocolitis (HAEC) is a complication of Hirschsprung disease (HSCR) that may occur both before or after surgery”
2) The statement, “We aimed to compare the incidence of HAEC following TSLPT and TEPT in HSCR patients...” may better read: “We aimed to compare the incidence of HAEC following TSLPT versus TEPT in HSCR patients…”
3) The statement, “We retrospectively reviewed the medical records of HSCR patients who underwent TSLPT and TEPT at our institution between 2018 and 2023” may better read: “We retrospectively reviewed the medical records of HSCR patients who underwent either TSLPT or TEPT at our institution between 2018 and 2023”
4) The word “frequency” in this statement and in several other places in the manuscript, have been used interchangeably with “incidence” which promotes ambiguity: The frequency of HAEC in TSLPT and TEPT was 24.4% and 6.9%, respectively. The authors need to clarify this. Most times “Frequency” is used to denote “absolute frequency” which is presented as counts. ‘Relative frequency’ is a fraction of the total which the authors maybe trying to present. ‘Proportion’ is still an appropriate term.
5) The authors need to take note of this statement written in the abstract, results etc: “These differences almost reached a significant level (p=0.056)”. It is necessary for the authors to state that the difference between the incidence of HAEC in TSLPT vs TEPT did not reach statistical significance. The statement, “almost reached a significant level (p=0.056)” may not be scientifically acceptable and its interpretation should be that of an insignificant finding.
6) The use of “prognostic factors” in the abstract and other sections of the manuscript is confusing. Prognostic factors are factors that predict the long term outcome/progression of a disease. Do the authors rather mean risk/predictor factors, which are more appropriate terms. If the authors actually mean prognostic factors then they should discuss more about these prognostic factors in the introduction/ discussion section. The authors should note the difference between risk factors, predictive factors, associated factors, prognostic factors and apply the terminology appropriately.
7) This statement written in the abstract and in line 193: “Subsequently, multivariate analysis indicated that albumin level is a strong prognostic factor for HAEC in HSCR patients following TEPT (p=0.047).” does not seem to be completely true, judging by the result obtained and presented in table 2. The confidence interval is showing it may rather be a weak predictor (the CI is wide and very close to 1.0)
8) These two sentences in the abstract are contradictory: “The frequency of HAEC in TSLPT and TEPT was 24.4% and 6.9%, respectively.” Vs “In conclusion, our study suggests that the incidence of HAEC tends to be higher following TEPT compared to TSLPT.”
9) This statement: “Furthermore, it emphasizes the importance of routinely monitoring albumin levels postoperatively to prevent HAEC in children with HSCR”, appears to over-generalize since the albumin levels was only significant in the TEPT group.

**Introduction:**

10) Since HAEC is the central outcome of interest, a bit more discussion of its pathophysiology or why it differs between techniques would be appropriate.

**Materials and Methods:**

11) Lines 89-90: “Data on HAEC in patients who underwent TSLPT and TEPT between 2018 and 2023...” may better read: “Data on HAEC in patients who underwent TSLPT or TEPT between 2018 and 2023…”
12) Two cut-off values, i.e., ≥4 and ≥10, were applied. The authors should indicate clearly what these cut-offs mean.
13) The authors need to clarify this sentence: Before participating, each patient's parent completed a written informed consent form. Was the consent obtained before the surgeries or obtained for the sole purpose of the present study.
14) “The data were accessed for research purposes from 02/10/2023 to 02/02/2024” This should better be taken to the data collection section and clarified as such.
15) The sentence: “Authors had access to information that could identify individual participants” may raise ethical concerns unless you clarify how privacy was protected (e.g., anonymized before analysis).
16) Lines 130-132: Nutritional status was assessed based on body mass index (BMI) and categorized as underweight (95th percentile) should be brought up close to other demographics ie after age classification on line 126.
17) Also, the BMI chart that was used for the percentile classification should be indicated. For example, CDC chart, WHO chart.
18) Table 1 and 2: “Diarrhea with an explosive tool.” Do the authors mean “Diarrhea with an explosive stool” instead.

Sample Size and Sampling Method:

19) The sample size calculation does not seem to be appropriate. The calculation using the equation presented, gives 47.4 instead of 68.
20) The authors wrote, “Substituting these values into the formula yielded… resulting in an estimated sample size of 68.” The authors however used sample size of 70. An explanation should be added. For example, the calculated sample size was 47 however all eligible cases were included which yielded a final sample size of 70. Whatever value is presented in this section should tally with the final sample size used.
21) “Chi-square or Fisher’s exact tests was applied to compare the frequency of HAEC between the TSLPT and TEPT groups”. This is vague. The authors should state the conditions where either Chi-square or Fisher’s exact tests were applied.

**Results:**

22) Table 2,3 For the last column p-value^a^, the authors may need to indicate (a) beside ones that were Fisher’s exact or if they choose, beside ones for chi square, and label beneath the table what the ones with (a) indicate, not both tests.
23) Authors should consider reorganizing table 2 for better flow. Demographics like sex, age and nutritional status should come before Aganglionosis type and other factors.
24) The authors wrote: “The frequency of HAEC in TSLPT and TEPT was 24.4% and 6.9%, respectively, using a cut-off of ≥4.” What about the proportion of HAEC in TSLPT and TEPT using a cut-off of ≥10 for completion?
25) In reporting the findings on line 179-182, authors should present the significant finding before the insignificant ones.
26) Looking at table 3 for instance, the total numbers for each of the variables do not round up to 70 as compared to table 1. For example, the variable “sex” total number in the Cut-off value ≥4 and Cut-off value ≥10 for both HAEC and Non-HAEC in the males and females categories is = 1 +18+ 1+ 9 + 1 + 18 + 0+ 10 = 58 and not 70. This is same for other variables. Authors should clarify the inconsistency in the total values.
27) Authors should interpret the multivariate analysis (logistic regression) using the odds ratio and confidence interval (i.e. risk). There was no interpretation of this in the entire manuscript. It may not be so appropriate to interpret logistic regression using p value alone.

**Discussion:**

28) Line 257 - 258: “The primary aim of this study was to evaluate the most appropriate surgical technique for the early prevention and management of HAEC”. This statement may not be appropriate as it is different from the primary aim of the present study which is “to analyze the difference in the incidence of Hirschprug-associated enterocolitis following Transanal endorectal pull through (TEPT) compared to transanal Swenson-like pull-through (TSLPT).”
29) Line 280 under Conclusions: Our study shows that the trend of HAEC is higher following TEPT than TSLPT. The authors should note that the present study did not examine trend, but rather examined the incidences.
30) Additionally, if they state the incidence of HAEC is higher following TEPT than TSLPT, they should add, “although it did not reach statistical significance.
31) Because hypoalbuminemia was only significant for the TEPT group at the cut-off of ≥4, it should be recognized as a potential risk factor not a definite prognostic factor.
32) Under the References section; Authors should cross-check the referencing style for appropriate use of upper case(Capital letters) in the titles and Journal names.
33) Additionally, the references should maintain same style. For example, to match this style: 2014;9(5):264-269 (if it is the chosen/accepted style)

**Reference 5:** 2022;17(1):150–4.

**Reference 7:** 2003; 238(4):569–76

**Reference 22:** 2023;15;10:1055128.
