# Supplementary material for: Does surgical approach affect Hirschsprung-associated enterocolitis risk? A comparison between transanal Swenson-like and endorectal pull-throughs
Source: PLoS One. 2026 Jan 8;21(1):e0340813. doi: 10.1371/journal.pone.0340813 (PMC12782437; doi:10.1371/journal.pone.0340813)
Supplement: Attachment — Submitted filename: Response Letter_AzZahra.docx [file pone.0340813.s004.docx]

**Response to Reviewers and Editors**

**Manuscript (Revised) Title:** Does surgical approach affect Hirschsprung-associated enterocolitis risk? A comparison between transanal Swenson-like and endorectal pull-throughs

**Reference #:** Submission ID PONE-D-25-45077

**Date:**

Dear Editorial Office, PLOS One,

We are grateful for the chance to revise our manuscript based on the valuable feedback from the reviewers and editors. We appreciate your constructive comments, which have enabled us further to enhance the quality and clarity of our paper. We have thoroughly addressed each point and revised the manuscript accordingly. The changes are listed in the revision section below.

Please find our point-by-point responses to the comments, with files consisting of:

- A marked-up copy of the manuscript that highlights changes made to the original version labeled '**Revised Manuscript with Track Changes**'.
- An unmarked version of the revised paper without tracked changes, labeled '**Manuscript'**.

**Editorial Comments:** When submitting your revision, we need you to address these additional requirements.

1. **Comment:** Please ensure that your manuscript meets PLOS ONE's style requirements, including those for file naming. The PLOS ONE style templates can be found at

- https://journals.plos.org/plosone/s/file?id=wjVg/PLOSOne_formatting_sample_main_body.pdf and
- https://journals.plos.org/plosone/s/file?id=ba62/PLOSOne_formatting_sample_title_authors_affiliations.pdf
  - **Response:** Thank you for this important reminder. We have carefully reviewed and revised the manuscript to ensure full compliance with *PLOS ONE*’s formatting and submission requirements. This includes adjustments to title page structure, author affiliations, heading levels, reference formatting, and figure/table legends according to the official *PLOS ONE* style templates. We have also ensured that all submitted files follow the required *PLOS ONE* file naming conventions.
  - **Changes:** The manuscript and all associated submission files (main text, figures, tables, and supplementary materials) have been reformatted to conform with *PLOS ONE*’s style and file-naming guidelines as outlined in the provided templates.

2. **Comment:** Please provide additional details regarding participant consent. In the ethics statement in the Methods and online submission information, please ensure that you have specified (1) whether consent was informed and (2) what type you obtained (for instance, written or verbal, and if verbal, how it was documented and witnessed). If your study included minors, state whether you obtained consent from parents or guardians. If the need for consent was waived by the ethics committee, please include this information.

If you are reporting a retrospective study of medical records or archived samples, please ensure that you have discussed whether all data were fully anonymized before you accessed them and/or whether the IRB or ethics committee waived the requirement for informed consent. If patients provided informed written consent to have data from their medical records used in research, please include this information.

- **Response:** Thank you for this important comment. We have revised the *Ethics Statement* in the *Methods* section and the online submission form to provide detailed information regarding participant consent. Written informed consent was obtained from the parents or legal guardians of all pediatric participants for the use of their anonymized medical data for research purposes. The consent process was conducted in accordance with institutional and ethical standards, and all data were fully anonymized prior to analysis.
- **Changes:** The protocol of this retrospective study was reviewed and approved by the Medical and Health Research Ethics Committee (MHREC), Faculty of Medicine, Public Health, and Nursing, Universitas Gadjah Mada, Yogyakarta, Indonesia (Approval number: KE/FK/0937/EC). Written informed consent was obtained from the parents or legal guardians of all participating patients, permitting the use of anonymized medical data for research purposes. All data were fully anonymized before access and analysis. The study was conducted in accordance with the principles of the Declaration of Helsinki and institutional ethical guidelines.

3. **Comment:** Your ethics statement should only appear in the Methods section of your manuscript. If your ethics statement is written in any section besides the Methods, please delete it from any other section.
   - **Response:** Thank you for the clarification. We have revised the manuscript accordingly. The *Ethics Statement* and *Consent Information* have been moved from the section after the Conclusion to the Methods section, in compliance with *PLOS ONE*’s formatting and reporting requirements. No ethical or consent information now appears outside of the Methods section.
   - **Changes:** The protocol of this retrospective study was reviewed and approved by the Medical and Health Research Ethics Committee (MHREC), Faculty of Medicine, Public Health, and Nursing, Universitas Gadjah Mada, Yogyakarta, Indonesia (Approval number: KE/FK/0937/EC). Written informed consent was obtained from the parents or legal guardians of all participating patients, permitting the use of anonymized medical data for research purposes. All data were fully anonymized before access and analysis. The study was conducted in accordance with the principles of the Declaration of Helsinki and institutional ethical guidelines.
4. **Comment:** If the reviewer comments include a recommendation to cite specific previously published works, please review and evaluate these publications to determine whether they are relevant and should be cited. There is no requirement to cite these works unless the editor has indicated otherwise.
   - **Response:** Thank you for the reminder. We have carefully reviewed all the suggested references provided by the reviewers. Relevant and appropriate citations have been incorporated into the manuscript to strengthen the background and discussion sections. At the same time, non-relevant works have been excluded to maintain focus on our study objectives. All newly added citations have been formatted in accordance with *PLOS ONE* reference style requirements.

**Reviewer #1 Comments:**

Does surgical approach affect Hirschsprung-associated enterocolitis risk? A comparison between transanal Swenson-like and endorectal pull-throughs

General statement:

The study by Azzahra et al. aimed to analyze the difference in the incidence of Hirschsprung-associated enterocolitis following Transanal endorectal pull-through (TEPT) compared to transanal Swenson-like pull-through (TSLPT). The application of Chi square test of independence/ Fisher’s exact test, and Multivariate analysis (logistic regression) to identify independent predictors of HAEC are appropriate for this study.

However, the following comments should be taken note of:

**Abstract:** This section needs to be rewritten:

1. The statement, “Hirschsprung-associated enterocolitis (HAEC) is a complication of Hirschsprung disease (HSCR) that occurs before and after surgery.” May be changed to “Hirschsprung-associated enterocolitis (HAEC) is a complication of Hirschsprung disease (HSCR) that may occur both before or after surgery”

- **Response:** Thank you for the valuable comment. We have revised the sentence to improve clarity and to reflect better that Hirschsprung-associated enterocolitis (HAEC) can occur either before or after surgical treatment.
- **Changes:** Hirschsprung-associated enterocolitis (HAEC) is a complication of Hirschsprung disease (HSCR) that may occur both before or after surgery.

2. The statement, “We aimed to compare the incidence of HAEC following TSLPT and TEPT in HSCR patients...” may better read: “We aimed to compare the incidence of HAEC following TSLPT versus TEPT in HSCR patients…”

- **Response:** Thank you for your helpful suggestion. We agree with this recommendation and have revised the sentence accordingly to enhance clarity and readability.
- **Changes:** We aimed to compare the incidence of HAEC following TSLPT versus TEPT in HSCR patients.

3. The statement, “We retrospectively reviewed the medical records of HSCR patients who underwent TSLPT and TEPT at our institution between 2018 and 2023” may better read: “We retrospectively reviewed the medical records of HSCR patients who underwent either TSLPT or TEPT at our institution between 2018 and 2023”

- **Response:** Thank you for this valuable suggestion. We agree with the reviewer’s comment and have revised the sentence to accurately indicate that each patient underwent one of the two surgical procedures.
- **Changes:** We retrospectively reviewed the medical records of HSCR patients who underwent either TSLPT or TEPT at our institution between 2018 and 2023.

4. The word “frequency” in this statement and in several other places in the manuscript, have been used interchangeably with “incidence” which promotes ambiguity: The frequency of HAEC in TSLPT and TEPT was 24.4% and 6.9%, respectively. The authors need to clarify this. Most times “Frequency” is used to denote “absolute frequency” which is presented as counts. ‘Relative frequency’ is a fraction of the total which the authors may be trying to present. ‘Proportion’ is still an appropriate term.

- **Response:** Thank you very much for this valuable suggestion. We agree with the reviewer’s comment and have revised the term “**frequency**” to “**proportion**” throughout the manuscript to more accurately describe the proportion of HAEC cases among patients in each surgical group. This change improves clarity and aligns with appropriate epidemiological terminology.

5. The authors need to take note of this statement written in the abstract, results etc: “These differences almost reached a significant level (p=0.056)”. It is necessary for the authors to state that the difference between the incidence of HAEC in TSLPT vs TEPT did not reach statistical significance. The statement, “almost reached a significant level (p=0.056)” may not be scientifically acceptable and its interpretation should be that of an insignificant finding.

- **Response:** Thank you very much for this important clarification. We agree with the reviewer’s comment. The wording has been revised throughout the manuscript (including in the Abstract and Results sections) to reflect that the difference was not statistically significant accurately.
- **Changes:**
- Abstract: “There was no statistically significant difference in the incidence of HAEC between the TSLPT and TEPT groups (p=0.056).”
- Results: “Although the incidence of HAEC was higher in the TSLPT group compared to the TEPT group, the difference did not reach statistical significance (p=0.056).”

6. The use of “prognostic factors” in the abstract and other sections of the manuscript is confusing. Prognostic factors are factors that predict the long-term outcome/progression of a disease. Do the authors rather mean risk/predictor factors, which are more appropriate terms? If the authors actually mean prognostic factors then they should discuss more about these prognostic factors in the introduction/ discussion section. The authors should note the difference between risk factors, predictive factors, associated factors, prognostic factors and apply the terminology appropriately.

- **Response:** Thank you for this insightful and constructive comment. We agree with the reviewer’s observation. In our study, the intent was to identify **predictor** **factors associated with the occurrence of HAEC**, not to evaluate long-term outcomes. Therefore, we have replaced the term *“prognostic factors”* with *“predictor factors”* throughout the manuscript to reflect the study objective more accurately.

7. This statement is written in the abstract and in line 193: “Subsequently, multivariate analysis indicated that albumin level is a strong prognostic factor for HAEC in HSCR patients following TEPT (p=0.047).” does not seem to be completely true, judging by the result obtained and presented in table 2. The confidence interval is showing it may rather be a weak predictor (the CI is wide and very close to 1.0)

- **Response:** Thank you very much for this important observation. We agree with the reviewer’s assessment. The wording has been revised to accurately reflect the statistical findings and to avoid overstating the strength of association. The revised version now indicates that albumin level was **significantly associated** with HAEC rather than being described as a *strong prognostic factor*. Additionally, we have replaced *“prognostic factor”* with *“risk factor”* throughout the manuscript, in accordance with an earlier reviewer comment.
- **Changes:**
  - Abstract: “Multivariate analysis indicated that low albumin level was significantly associated with the occurrence of HAEC in HSCR patients following TEPT (p=0.047).”
  - Results: “On multivariate analysis, low albumin level remained significantly associated with HAEC occurrence after TEPT (p=0.047; 95% CI: 0.010–0.970), although the wide confidence interval suggests a weak predictive value.”

8. These two sentences in the abstract are contradictory: “The frequency of HAEC in TSLPT and TEPT was 24.4% and 6.9%, respectively.” Vs “In conclusion, our study suggests that the incidence of HAEC tends to be higher following TEPT compared to TSLPT.”

- **Response:** Thank you for your valuable comment. We appreciate your careful reading of the abstract. The inconsistency you noted resulted from a typographical error in the reported proportions of HAEC between the TSLPT and TEPT groups. The correct statement should read: *“The proportion of HAEC in TEPT and TSLPT was 24.4% and 6.9%, respectively.”* We have corrected this error in the revised manuscript. With this correction, the conclusion remains accurate and is supported by the corrected data.
- **Changes:** The proportion of HAEC in TEPT and TSLPT was 24.4% and 6.9%, respectively.

9. This statement: “Furthermore, it emphasizes the importance of routinely monitoring albumin levels postoperatively to prevent HAEC in children with HSCR”, appears to over-generalize since the albumin levels was only significant in the TEPT group.

- **Response:** Thank you for this helpful comment. We agree that the original sentence over-generalized the role of albumin. Since hypoalbuminemia was found to be significant **only within the TEPT subgroup**, we have revised the statement to accurately reflect that postoperative albumin monitoring may serve as a **potential risk indicator** for HAEC specifically in TEPT patients, rather than a universal factor for all HSCR patients.
- **Changes:** Monitoring albumin levels postoperatively may be considered as a potential risk indicator for diagnosing HAEC in patients undergoing surgery especially TEPT, as hypoalbuminemia showed significance only within this surgical subgroup.

**Introduction:**

10. Since HAEC is the central outcome of interest, a bit more discussion of its pathophysiology or why it differs between techniques would be appropriate.

- **Response:** Thank you for this insightful suggestion. We agree that expanding the discussion on the pathophysiology of HAEC and its potential relationship with different surgical techniques will improve the clarity and context of our findings. We have added a more detailed explanation in the Discussion section addressing the underlying mechanisms—such as impaired enteric nervous system function, altered gut motility, dysbiosis, and mucosal immune dysregulation—and how different pull-through techniques may influence these factors. This addition strengthens the rationale for why HAEC incidence may vary between TSLPT and TEPT.
- **Changes:** HAEC is hypothesized to arise from a multifactorial interplay of decreased enteric nervous system function, altered gut motility, dysbiosis, and dysregulation of mucosal immunity [8,16,17]. Variations in surgical techniques may influence these parameters differently. For example, differences in the extent of aganglionic segment excision, anastomotic tension, and postoperative colonic motility restoration may contribute to the varying susceptibility to HAEC between procedures [16,17]. These physiological distinctions may partly explain the differing proportions of HAEC observed between TSLPT and TEPT in our cohort.
- **References:**

8.Gershon EM, Rodriguez L, Arbizu RA. Hirschsprung's disease associated enterocolitis: A comprehensive review. *World J Clin Pediatr* 2023; 12(3): 68-76 [PMID: [37342453](http://www.ncbi.nlm.nih.gov/pubmed/37342453) DOI: [10.5409/wjcp.v12.i3.68](https://dx.doi.org/10.5409/wjcp.v12.i3.68)]

16.Ziogas IA, Kuruvilla KP, Fu M, Gosain A. Hirschsprung-associated enterocolitis: a comprehensive review. *World J Pediatr Surg*. 2024;7(3):e000878. Published 2024 Oct 14. doi:10.1136/wjps-2024-000878

17.Gao T, Xu W, Sheng Q, Xu T, Wu W, Lv Z. Clinical outcomes and risk factors for postoperative complications in children with Hirschsprung's disease. *Am J Transl Res*. 2022;14(7):4830-4837. Published 2022 Jul 15.

**Materials and Methods:**

11. Lines 89-90: “Data on HAEC in patients who underwent TSLPT and TEPT between 2018 and 2023...” may better read: “Data on HAEC in patients who underwent TSLPT or TEPT between 2018 and 2023…”

- **Response:** Thank you for the helpful suggestion. We agree with the reviewer’s recommendation and have revised the sentence to ensure grammatical correctness and clarity.
- **Changes**: “Data on HAEC in patients who underwent TSLPT or TEPT between 2018 and 2023 were collected…”

12. Two cut-off values, i.e., ≥4 and ≥10, were applied. The authors should indicate clearly what these cut-offs mean.

- **Response:** Thank you for this very important comment. We agree that further clarification and transparency are needed regarding the use of the two cut‐off values and the scoring system for HAEC in our study. We have revised the Methods section to include:
  - A brief explanation of the original scoring systems developed in the literature and their diagnostic cut‐offs (≥10 and ≥4) as reported in prior work.
  - A clear statement that in our study we applied both cut‐off values (≥10 and ≥4) to explore how incidence/proportion of HAEC varied under each threshold.
  - A description of how the scores were assigned in our dataset: namely, we reviewed medical records of patients, extracted the presence/absence of each criterion in the score, and calculated the total score for each encounter. This scoring was performed by paediatric surgery fellows, and discrepancies were resolved by consensus with the senior author to ensure consistency.
  - A note on validation/reproducibility: We acknowledge that while the original scoring systems have been evaluated for sensitivity/specificity, our institution did not perform external validation of the scoring in a prospective manner, and we state this as a limitation.
  - These changes have been incorporated into the revised manuscript to improve clarity and reproducibility.
- **Changes:**
- *Methods Section – HAEC Scoring and Cut-offs:*

“Diagnosis of HAEC was determined using a clinical scoring system originally developed in prior literature (e.g., the Pastor score and the Frykman score), which incorporates history, physical examination, radiologic, and laboratory criteria. In the Pastor score, the original diagnostic cut‐off was ≥10 points; subsequently, other authors proposed that a lower cut‐off of ≥4 points increased sensitivity. In our study, we applied both cut-off values (≥10 and ≥4) to the scoring of each patient encounter to compare how the proportion of HAEC would vary under each threshold. Scoring was performed by a pediatric surgeon who extracted the presence/absence of each criterion from the medical record; any discrepancies were resolved in consensus with the senior author.”

- *Discussion Section (revised paragraph):*

“We applied two cut‐off values (≥10 and ≥4) for HAEC diagnosis in our cohort. Prior studies have demonstrated that the higher threshold (≥10) yields high specificity but may miss milder cases [18], whereas the lower threshold (≥4) improves sensitivity and may capture earlier or milder episodes of HAEC [19,20]. By reporting both thresholds, we highlight how the incidence/proportion of HAEC varies depending on the diagnostic criteria used, and we recommend that future studies clearly state the cut‐off used and validate scoring systems in their own populations.”

*Limitation:* The scoring system has limited prospective validation, and inter-rater reliability was not formally measured in our study.

- **References:**

18.Pastor AC, Osman F, Teitelbaum DH, et al. Development of a standardized definition for Hirschsprung’s-associated enterocolitis: a Delphi analysis. J Pediatr Surg. 2009;44:251–6.

19.Frykman PK, Kim S, Wester T, et al. Critical evaluation of the Hirschsprung-associated enterocolitis (HAEC) score: a multicenter study of 116 children with Hirschsprung disease. J Pediatr Surg. 2018;53:708–17.

20.Gunadi, Sukarelawanto, A.V.R., Ritana, A. *et al.* Postoperative enterocolitis assessment using two different cut-off values in the HAEC score in Hirschsprung patients undergoing Duhamel and Soave pull-through. *BMC Pediatr* 20, 457 (2020). https://doi.org/10.1186/s12887-020-02360-x

13. The authors need to clarify this sentence: Before participating, each patient's parent completed a written informed consent form. Was the consent obtained before the surgeries or obtained for the sole purpose of the present study.

- **Response:** Thank you for the reminder. The informed consent was obtained specifically for the purpose of this study, not prior to the patients’ surgeries.
- **Changes:** We have revised the sentence to: “For this study, written informed consent was obtained from each patient’s parent prior to inclusion in the study.

14. “The data were accessed for research purposes from 02/10/2023 to 02/02/2024” This should better be taken to the data collection section and clarified as such.

- **Response:** Thank you for this helpful comment. We agree with the reviewer’s suggestion. The statement has been moved from the methods introduction to the **data collection section** for better organization and clarity. The wording has also been slightly revised to clearly indicate the data collection period.
- **Changes:** (Data Collection) Data were collected for research purposes between October 2, 2023, and February 2, 2024.

15. The sentence: “Authors had access to information that could identify individual participants” may raise ethical concerns unless you clarify how privacy was protected (e.g., anonymized before analysis).

- **Response:** Thank you for this helpful suggestion. Although the authors initially had access to identifiable information during data collection, all data were fully anonymized prior to analysis. Identifying variables (such as names, dates of birth, or medical record numbers) were replaced with study codes and only de-identified datasets were used in the statistical analysis. No identifiable information was stored, analyzed, or reported in the manuscripts.
- **Changes:** Authors had access to information that could identify anonymized individual participants during or after data collection. Identifying variables (such as names, dates of birth, or medical record numbers) were replaced with study codes, and only de-identified datasets were used in the statistical analysis. No identifiable information was stored, analyzed, or reported in the manuscripts.

16. Lines 130-132: Nutritional status was assessed based on body mass index (BMI) and categorized as underweight (95th percentile) should be brought up close to other demographics i.e. after age classification on line 126.

- **Response:** Thank you for this helpful suggestion. We agree with the reviewer’s comment and have repositioned the sentence on nutritional status to immediately follow the age classification in the demographic section. This revision improves the logical flow and readability of the Methods section.
- **Changes:** The sentence now appears directly after the description of age classification: “Nutritional status was assessed based on body mass index (BMI) and categorized as underweight (<5th percentile), normal (5th–95th percentile), and overweight (>95th percentile).”

17. Also, the BMI chart that was used for the percentile classification should be indicated. For example, the CDC chart, WHO chart.

- **Response:** Thank you for the insightful observation. In the manuscript, we used the WHO BMI classification. We agree that this should have been stated more clearly. As a revision, we have added this detail to the manuscript.
- **Changes:** Nutritional status was assessed based on body mass index (BMI) and categorized as underweight (<5th percentile), ideal (5th–85th percentile), overweight (85th–95th percentile), or obese (>95th percentile) using the WHO Chart
- **References:** <https://www.who.int/tools/child-growth-standards/standards/weight-for-length-height>

18. Table 1 and 2: “Diarrhea with an explosive tool.” Do the authors mean “Diarrhea with an explosive stool” instead.

- **Response:** Thank you for noticing this typographical error. We appreciate the reviewer’s careful reading. The word *“tool”* was indeed a typographical mistake and has been corrected to *“stool”* in both Table 1 and Table 2.
- **Changes:** Revised text (Table 1 and Table 2): “Diarrhea with an explosive stool.”

**Sample Size and Sampling Method:**

19. The sample size calculation does not seem to be appropriate. The calculation using the equation presented, gives 47.4 instead of 68.

- **Response:** Thank you for your careful assessment. You are correct that, using the parameters presented in the equation, the calculated sample size is 47.4, not 68. We have recalculated and confirmed that the correct minimum required sample size is 47.4 (rounded up to 48). We apologize for this numerical error.
- **Changes:** In the *Sample Size and Sampling Method* section, we have revised the final sentence as follows: “Substituting these values into the formula yielded: 𝑛 = (1.96)² × 0.231 × (1−0.231) / (0.12)², resulting in an estimated minimum sample size of 47.4, which was rounded up to 48.”

20. The authors wrote, “Substituting these values into the formula yielded… resulting in an estimated sample size of 68.” The authors however used a sample size of 70. An explanation should be added. For example, the calculated sample size was 47 however all eligible cases were included which yielded a final sample size of 70. Whatever value is presented in this section should tally with the final sample size used.

- **Response:** Thank you for the helpful suggestion. The discrepancy occurred because all eligible cases during the study period were included in the analysis. Although the calculated minimum required sample size was 47, we used all 70 eligible patients to maximize statistical power and ensure comprehensive representation of our institutional data. We have now clarified this in the revised manuscript.
- **Changes:** We have revised the paragraph in the *Sample Size and Sampling Method* section as follows: “Using the single-proportion formula, the minimum required sample size was calculated to be 47.4 (rounded to 48). However, because all eligible Hirschsprung disease cases with complete medical records from 2018 to 2023 were included, the final sample size for this study was 70.”

21. “Chi-square or Fisher’s exact tests were applied to compare the frequency of HAEC between the TSLPT and TEPT groups”. This is vague. The authors should state the conditions where either Chi-square or Fisher’s exact tests were applied.

- **Response:** Thank you for pointing this out. In our analysis, all bivariate comparisons of categorical variables—including the comparison of HAEC frequency between the TSLPT and TEPT groups—were conducted using the Chi-square test. Fisher’s exact test was not ultimately required because all contingency table cells met the minimum expected frequency assumption for Chi-square analysis. We have clarified this in the manuscript.
- **Changes:** The sentence has been revised to: “Chi-square tests were applied to compare the proportion of HAEC between the TSLPT and TEPT groups.”

**Results:**

22. Table 2,3 For the last column p-values, the authors may need to indicate (a) beside ones that were Fisher’s exact or if they choose, beside ones for chi square, and label beneath the table what the ones with (a) indicate, not both tests.

- **Response:** Thank you for pointing this out. In our analysis, all bivariate comparisons of categorical variables including the comparison of HAEC proportion between the TSLPT and TEPT groups were conducted using the Chi-square test. Fisher’s exact test was not required because all contingency table cells met the minimum expected frequency assumptions for Chi-square analysis. With this, no (a) notation is needed for individual p-values in the tables.
- **Changes:** In the Statistical Analysis section:
- *“*Chi-square tests were applied to compare categorical variables, as all contingency table cells met the minimum expected frequency required for Chi-square analysis.”
- The following clarification has been added to the footnotes of Table 2 and Table 3: “Analyzed using the Chi-square test.”
- Removed any previous wording that stated “Chi-square or Fisher’s exact test.

23. Authors should consider reorganizing Table 2 for better flow. Demographics like sex, age and nutritional status should come before Aganglionosis type and other factors.

- **Response:** Thank you for this helpful suggestion. We agree that reorganizing Table 2 will improve readability and logical flow. In the revised manuscript, we have restructured Table 2 so that demographic variables (sex, age, nutritional status) are presented first, followed by aganglionosis type, clinical factors, and laboratory findings.

24. The authors wrote: “The frequency of HAEC in TSLPT and TEPT was 24.4% and 6.9%, respectively, using a cut-off of ≥4.” What about the proportion of HAEC in TSLPT and TEPT using a cut-off of ≥10 for completion?

- **Response:** Thank you for this useful comment. We agree that presenting both cut-off values provides a more complete picture of HAEC proportions across surgical groups. We have now added the corresponding proportions for the ≥10 cut-off value. This revision improves clarity and allows readers to compare HAEC rates under both diagnostic thresholds.
- **Changes:** Using a cut-off of ≥4, the proportion of HAEC in TEPT and TSLPT was 24.4% and 6.9%, respectively. When applying the cut-off of ≥10, the proportion decreased to 9.8% in TEPT and 3.4% in TSLPT.

25. In reporting the findings on line 179-182, authors should present the significant findings before the insignificant ones.

- **Response:** Thank you for this helpful suggestion. We have revised the Results section so that the significant finding—postoperative albumin level in the TEPT group—is presented before the non-significant variables. This improves the clarity and emphasis of the results, as recommended.
- **Changes:** Postoperative albumin level was the only variable that showed a significant association with HAEC in the TEPT group (p = 0.03). In contrast, other factors, including sex, age at surgery, nutritional status, and postoperative hemoglobin level, did not demonstrate significant associations with HAEC in either surgical group (Tables 3 and 4).

26. Looking at table 3 for instance, the total numbers for each of the variables do not round up to 70 as compared to table 1. For example, the variable “sex” total number in the Cut-off value ≥4 and Cut-off value ≥10 for both HAEC and Non-HAEC in the males and females categories is = 1 +18+ 1+ 9 + 1 + 18 + 0+ 10 = 58 and not 70. This is the same for other variables. Authors should clarify the inconsistency in the total values.

- **Response:** Thank you for highlighting this point. The totals in Tables 3 and 4 do not sum to 70 because the analyses in these tables were performed **separately for the TSLPT group (n=29)** and **the TEPT group (n=41)**. Therefore, each table presents subgroup analyses rather than the full sample. In Table 3 (TSLPT group), all variable totals are based on **n=29**, while in Table 4 (TEPT group), totals are based on **n=41**. We have added clarifying statements in the manuscript and table footnotes to avoid further confusion.
- **Changes:** Tables 3 and 4 present subgroup analyses stratified by surgical technique. Table 3 includes only TSLPT patients (n=29), and Table 4 includes only TEPT patients (n=41). Thus, totals in these tables correspond to the subgroup sample sizes rather than the overall sample of 70 patients.

27. Authors should interpret the multivariate analysis (logistic regression) using the odds ratio and confidence interval (i.e. risk). There was no interpretation of this in the entire manuscript. It may not be so appropriate to interpret logistic regression using p-value alone.

- **Response:** Thank you for this important comment. We agree that logistic regression results should be interpreted using odds ratios and confidence intervals rather than p-values alone. In response, we have revised the Results and Discussion sections to include explicit interpretation of the multivariate logistic regression model, incorporating both the odds ratio (Exp[B]) and its 95% confidence interval. We now describe which factors increased or decreased the odds of HAEC and clarify the precision of these estimates using the confidence intervals. These revisions strengthen the clarity and scientific rigor of our statistical interpretation.
- **Changes:**
  - Results: On multivariate analysis, low albumin level remained significantly associated with HAEC occurrence after TEPT (p=0.047; 95% CI: 0.010–0.970), although the wide confidence interval suggests a weak predictive value. Lower postoperative albumin levels were associated with increased odds of HAEC (OR = 0.096, 95% CI: 0.010–0.970, p = 0.047), indicating that a decrease in albumin may increase the likelihood of HAEC. Other variables, including sex (OR = 0.245, 95% CI: 0.032–1.896), nutritional status (OR = 0.894, 95% CI: 0.056–14.353), and postoperative hemoglobin level (OR = 0.137, 95% CI: 0.009–2.038), did not show statistically significant associations with HAEC, and their wide confidence intervals suggest limited precision and a lack of strong predictive value.
  - Discussion: The multivariate analysis demonstrated that postoperative hypoalbuminemia was the strongest risk indicator for HAEC in the TEPT group, with an odds ratio of 0.096 (95% CI: 0.010–0.970). This suggests that lower albumin may increase vulnerability to HAEC, although the wide confidence interval indicates moderate uncertainty in the magnitude of this effect. In contrast, sex, nutritional status, and postoperative hemoglobin levels showed no significant associations with HAEC, and their broad confidence intervals highlight limited predictive value. These findings underscore that while hypoalbuminemia may be clinically relevant in the TEPT cohort, the evidence does not support these other factors as meaningful predictors in our population.

**Discussion:**

28. Line 257 - 258: “The primary aim of this study was to evaluate the most appropriate surgical technique for the early prevention and management of HAEC”. This statement may not be appropriate as it is different from the primary aim of the present study which is “to analyze the difference in the incidence of Hirschsprung-associated enterocolitis following Transanal endorectal pull through (TEPT) compared to transanal Swenson-like pull-through (TSLPT).”

- **Response:** Thank you for this valuable comment. We agree with the reviewer’s observation. The original statement inaccurately conveyed the study’s objective. It has been revised to align with the stated primary aim presented in the abstract and methods section, ensuring consistency throughout the manuscript.
- **Changes**: “The primary aim of this study was to analyze the difference in the incidence of HAEC following TEPT compared to TSLPT.”

29. Line 280 under Conclusions: Our study shows that the trend of HAEC is higher following TEPT than TSLPT. The authors should note that the present study did not examine trends, but rather examined the incidences.

- **Response:** Thank you for the observation. We agree that the term “trend” is inaccurate, as our study assessed *incidence* (proportion) rather than temporal trends. We have revised the sentence accordingly.
- **Changes:** The sentence in the Conclusions has been updated to: “Our study shows that the incidence of HAEC is higher following TEPT than TSLPT.”

30. Additionally, if they state the incidence of HAEC is higher following TEPT than TSLPT, they should add, “although it did not reach statistical significance.

- **Response:** Thank you for this important comment. We agree with the reviewer’s recommendation. We have revised the sentence in the Conclusions to indicate that although the incidence of HAEC was higher following TEPT compared to TSLPT, this difference did not reach statistical significance.
- **Changes:** “Our study shows that the incidence of HAEC is higher following TEPT than TSLPT, although this difference did not reach statistical significance.”

31. Because hypoalbuminemia was only significant for the TEPT group at the cut-off of ≥4, it should be recognized as a potential risk factor not a definite prognostic factor.

- **Response:** Thank you for this important clarification. We agree with the reviewer’s assessment. The statement has been revised to indicate that hypoalbuminemia represents a **potential risk factor**, rather than a **definite prognostic factor**, as the finding was only significant for the TEPT group and the statistical strength was limited. The terminology has been adjusted accordingly throughout the manuscript to accurately reflect this interpretation.
- **Changes:** Conclusion: “Notably, hypoalbuminemia appeared as a potential risk factor for HAEC in patients undergoing TEPT; however, given the limited statistical significance and confidence interval range, this association should be interpreted with caution.”

**References**

32. Under the References section; Authors should cross-check the referencing style for appropriate use of upper case(capital letters) in the titles and journal names.

- **Response:** Thank you for this valuable comment. We have carefully reviewed and corrected all references to ensure consistency with the journal’s referencing style. The use of upper case letters in article titles and journal names has been standardized according to the journal’s formatting guidelines.
- **Changes:** All references have been edited to reflect proper capitalization (e.g., sentence case for article titles and title case for journal names) in accordance with the journal’s reference style requirements.

33. Additionally, the references should maintain the same style. For example, to match this style: 2014;9(5):264-269 (if it is the chosen/accepted style)

Reference 5: 2022;17(1):150–4.

Reference 7: 2003; 238(4):569–76

Reference 22: 2023;15;10:1055128.

- **Response:** Thank you for this helpful observation. We have thoroughly reviewed all references to ensure uniformity in citation formatting. The references have been edited to follow the consistent style requested by the reviewer, particularly for publication year, volume, issue, and page number formatting (e.g., *2014;9(5):264–269*).
- **Changes:** All references have been reformatted for consistency.

**Reviewer #2 Comments:**

I have reviewed the manuscript titled “Does surgical approach affect Hirschsprung-associated enterocolitis risk? A comparison between transanal Swenson-like and endorectal pull-throughs" (PONE-D-25-45077).

There are some important comments that should be considered.

1. The **Introduction** cites recent studies from 2023 and 2024 (references 9 and 10) suggesting that the transanal Swenson-like pull-through (TSLPT) technique may reduce mechanical and functional complications, including Hirschsprung-associated enterocolitis (HAEC). However, immediately following this, the manuscript references a much older study from 2011 (reference 11) to emphasize that despite surgical advances, HAEC remains a significant risk for morbidity and mortality. This sequence creates a temporal disconnect in the justification of the study, as the more recent references imply ongoing advancements, while the older reference suggests persistent high risk.

- **Response:** Thank you for highlighting this point. We agree that the chronological presentation of the literature may cause a temporal disconnect. To address this, we have reorganized the introduction so that the older foundational study is presented first to establish the historical context of HAEC as a persistent clinical challenge. We then introduce the more recent studies (2023–2024), which explore improvements in surgical techniques such as TSLPT. This revised order presents a more logical progression: from longstanding concern regarding HAEC to current efforts aimed at reducing postoperative complications. We have updated the relevant paragraph in the Introduction accordingly.
- **Changes:** HAEC has long been recognized as a major source of postoperative morbidity and mortality in patients with Hirschsprung disease, as demonstrated in earlier studies that reported significant risk despite surgical advancements [5]. Despite progress in surgical management, HAEC continues to pose a significant risk for morbidity and mortality [5]. The incidence of HAEC ranges from 1% to 10%, with the highest risk observed in neonates before definitive surgery [5]. Patients with HSCR who develop HAEC during the neonatal period typically experience a hospital stay that is twice as long as those without this complication [6].

Several factors—such as timing of definitive surgery, nutritional status, and maternal characteristics—have been considered as contributors to the development of HAEC [9].

In recent years, emerging techniques such as the transanal Swenson-like pull-through (TSLPT) have shown promise in reducing mechanical and functional complications, including postoperative HAEC [13]. Recent studies suggested that the TSLPT technique may lower the risk of mechanical and functional complications, highlighting the urgency of evaluating and adopting the most effective surgical approach to improve patient outcomes, including HAEC [14,15].

Despite these developments, evidence remains limited, and the incidence of HAEC continues to vary widely across centers, underscoring the need for further investigation into factors contributing to its occurrence.

2. **Introduction**: Additionally, the manuscript cites an older reference from 1989 (reference 12) to support the claim that neonates with Hirschsprung disease who develop HAEC experience hospital stays twice as long as those without this complication. Given the age of this reference, it raises concerns about the currency and contextual relevance of this justification, especially when more recent studies have likely addressed hospital stay durations and morbidity associated with HAEC. The authors should consider including updated evidence to maintain the temporal consistency and strengthen the rationale of their study.

- **Response:** Thank you for this valuable suggestion. We agree that relying solely on a 1989 reference may not adequately reflect current clinical outcomes. In response, we have added more recent literature reporting prolonged hospitalization and increased morbidity associated with HAEC in the contemporary surgical era. These updated references provide stronger and more temporally relevant support for the statement. We have revised the Introduction accordingly and retained the 1989 study only as historical context.
- **Changes:** Patients with HSCR who develop HAEC during the neonatal period typically experience a hospital stay that is twice as long as those without this complication [6]. More recent studies, however, continue to demonstrate that HAEC is associated with longer hospitalization and increased morbidity in the current era of improved perioperative care and surgical techniques [7,8]. For example, one 2022 review noted that the mean duration of hospitalization for HAEC patients was 13 days (ranging from 6 to 29 days) and that medical management of HSCR children with HAEC is 2.5 times costlier than for those without HAEC [7]. A comprehensive review published in 2024 reinforces that HAEC is an important cause of morbidity and the leading cause of mortality in patients with HSCR [8]. These contemporary data reinforce that HAEC remains a clinically impactful complication despite advancements in management [8].
- **References:**

7.Lewit RA, Kuruvilla KP, Fu M, Gosain A. Current understanding of Hirschsprung-associated enterocolitis: Pathogenesis, diagnosis and treatment. *Semin Pediatr Surg*. 2022;31(2):151162. doi:10.1016/j.sempedsurg.2022.151162

8.Ziogas IA, Kuruvilla KP, Fu M, Gosain A. Hirschsprung-associated enterocolitis: a comprehensive review. *World J Pediatr Surg*. 2024;7(3):e000878. Published 2024 Oct 14. doi:10.1136/wjps-2024-000878

3. Based on the relevance of the prognostic factors analyzed—sex, aganglionosis type, nutritional status, age at surgery, and postoperative hemoglobin and albumin levels—it would strengthen the manuscript if the authors explicitly addressed and discussed these factors in the **discussion** section. Highlighting their importance and contextualizing the study findings within the framework of existing literature on these predictive factors would provide a clearer interpretation of the **results** and reinforce the study’s significance.

- **Response:** We thank the reviewer for this valuable and constructive suggestion. We agree that a more explicit discussion of the prognostic factors analyzed—namely sex—would strengthen the interpretation of our results. Aganglionosis type, age at surgery, nutritional status, postoperative hemoglobin, and albumin levels have already been discussed in the previous manuscript. Accordingly, we have revised the Discussion section to clearly address each factor and contextualize our findings with current evidence. These additions highlight the importance of the examined variables and clarify their potential impact on HAEC development following TSLPT and TEPT—the revised and expanded discussion of the manuscript.
- **Changes:**

Sex did not influence the proportion of HAEC. This aligns with several reports indicating that although HSCR is more common in males, sex itself does not seem to affect the risk of postoperative HAEC. A recent national cohort study exploring potential sex differences in HSCR outcomes found that the early clinical course of patients does not appear to depend on sex, with no significant difference in HAEC incidence between males and females [26]. Other literature reviews have similarly concluded that while some earlier, smaller studies suggested a possible link to female gender, the majority of evidence, including recent comprehensive analyses, points toward no significant association between sex and the risk of developing HAEC [27].

- **References:**

26.Le-Nguyen A, Righini-Grunder F, Piché N, Faure C, Aspirot A. Factors influencing the

incidence of Hirschsprung associated enterocolitis (HAEC). J Pediatr Surg 2019; 54(5):

959-63.

27.Söderström, L., Graneli, C., Hagelsteen, K. *et al.* Exploring potential sex differences in

Hirschsprung disease: a national cohort study of diagnostic patterns and early postoperative

outcome. *Pediatr Surg Int* 41, 309 (2025). <https://doi.org/10.1007/s00383-025-06213-5>

4. The authors have applied appropriate statistical methods, including sample size calculation based on a single-proportion formula and suitable use of Chi-square, Fisher’s exact, and logistic regression analyses. However, the relatively small sample sizes within each surgical subgroup (29 in TSLPT, 41 in TEPT) may limit the power to detect subtle differences, raising the possibility of type II error. It would strengthen the manuscript if the authors explicitly **acknowledge this limitation** and **consider providing post-hoc power analyses** to better contextualize non-significant results that may still be clinically relevant. Furthermore, a more detailed description of how missing data were handled and whether any sensitivity analyses were performed would improve confidence in the robustness of the findings. Overall, while the statistical approach is sound, caution should be exercised in interpreting results, particularly borderline findings, given the sample size and retrospective design constraints.
   - **Response:** Thank you for this valuable comment. We agree that the relatively small sample sizes in each surgical subgroup (TSLPT n=29; TEPT n=41) may limit the statistical power to detect subtle differences, increasing the possibility of a type II error. We have now explicitly acknowledged this limitation in the manuscript. As suggested by the reviewer, we have now performed the requested post hoc analyses and incorporated them into the revised manuscript. In addition, the complete post-hoc results have been included in the updated Supplementary Tables for clarity and completeness.

Additionally, we have clarified how missing data were handled. Only patients with complete medical records were included in the analysis, and no imputation was performed. Since the dataset did not include partially missing variables among the included cases, sensitivity analyses were not required. This information has been added to the Methods and Limitations sections.

- **Changes:**
- **Methods:** Only cases with complete medical records were included, and no imputation was performed. Because no partially missing variables were present in the included dataset, sensitivity analyses were not required.

Post-hoc analyses for categorical variables were performed using adjusted residuals following significant Chi-square tests; values ≥|2| were considered indicative of meaningful cell-level deviation.

**Result:** Significant post-hoc deviations were observed only for albumin in the TEPT ≥4 group, with hypoalbuminemia showing fewer-than-expected HAEC cases and normal albumin showing more. All other variables showed adjusted residuals <2 and were not significant (Supp. Table 2).

**Discussion:** Among all evaluated variables in post-hoc analyses, albumin level was the only factor showing a statistically significant deviation in HAEC distribution, observed in the TEPT group at a cut-off ≥4. Patients with normal albumin levels had more HAEC events than expected, whereas those with hypoalbuminemia had fewer. Although counterintuitive, this pattern may reflect selection bias or small-sample behavior rather than a true biological effect, as the association was not reproduced at other cut-off levels or in the TSLPT group. No consistent, significant findings were observed for sex, age at surgery, nutritional status, or hemoglobin level, suggesting that these variables may not meaningfully contribute to HAEC risk in the postoperative period.

- **References:**

Cohen J. *Statistical Power Analysis for the Behavioral Sciences.* 2nd ed. Lawrence Erlbaum Associates; 1988.

5. The manuscript's description of the HAEC scoring system lacks sufficient detail about the **specific diagnostic criteria**, including how the scores are determined and whether the scoring has been validated or has good reproducibility. As the diagnosis of HAEC is inherently challenging due to its non-specific signs and symptoms, providing a clear and detailed explanation of the scoring system would enhance the transparency and reproducibility of the study. Currently, the manuscript would benefit from elaborating on how the scores were assigned, who performed the scoring, and whether any measures were taken to ensure consistency across different raters or centers.

- **Response:** Thank you for this very important comment. We agree that further clarification and transparency are needed regarding the use of the two cut‐off values and the scoring system for HAEC in our study. We have revised the Methods section to include:
  - A brief explanation of the original scoring systems developed in the literature and their diagnostic cut‐offs (≥10 and ≥4) as reported in prior work.
  - A clear statement that in our study, we applied both cut‐off values (≥10 and ≥4) to explore how the incidence/proportion of HAEC varied under each threshold.
  - A description of how the scores were assigned in our dataset: namely, we reviewed medical records of patients, extracted the presence/absence of each criterion in the score, and calculated the total score for each encounter. The scores were assigned based on the physical examination performed by a pediatric surgeon when the patient presented to our department. The scoring was taken from a previous study: doi: [10.3389/fped.2021.705663](https://doi.org/10.3389/fped.2021.705663)
  - A note on validation/reproducibility: We acknowledge that although the original scoring systems have been evaluated for sensitivity/specificity, our institution did not conduct prospective external validation, which we state as a limitation.
  - These changes have been incorporated into the revised manuscript to improve clarity and reproducibility.
- **Changes:**
- Methods Section – HAEC Scoring and Cut-offs:

“Diagnosis of HAEC was determined using a clinical scoring system originally developed in prior literature (e.g., the Pastor score and the Frykman score), which incorporates history, physical examination, radiologic, and laboratory criteria [18,19]. In the Pastor score, the original diagnostic cut‐off was ≥10 points [18]; subsequently, other authors proposed that a lower cut‐off of ≥4 points increased sensitivity [19]. In our study, we applied both cut-off values (≥10 and ≥4) to the scoring of each patient encounter to compare how the proportion of HAEC would vary under each threshold. Scoring was performed by a pediatric surgeon who extracted the presence/absence of each criterion from the medical record [20]. The criteria included: explosive diarrhea, foul-smelling diarrhea, bloody diarrhea, abdominal distention, fever, lethargy, dilated bowel loops on imaging, decreased peripheral perfusion, leukocytosis, and shift to left. We acknowledge that the scoring system has limited prospective validation and that inter-rater reliability was not formally measured in our study—this is noted in the Limitations section.”

- Discussion Section:

“We applied two cut‐off values (≥10 and ≥4) for HAEC diagnosis in our cohort. Prior studies have demonstrated that the higher threshold (≥10) yields high specificity but may miss milder cases [18], whereas the lower threshold (≥4) improves sensitivity and may capture earlier or milder episodes of HAEC [19,20]. By reporting both thresholds, we highlight how the incidence/proportion of HAEC varies with the diagnostic criteria used, and we recommend that future studies clearly state the cut-off used and validate scoring systems in their own populations.”

- **References:**

18.Pastor AC, Osman F, Teitelbaum DH, et al. Development of a standardized definition for Hirschsprung’s-associated enterocolitis: a Delphi analysis. J Pediatr Surg. 2009;44:251–6.

19.Frykman PK, Kim S, Wester T, et al. Critical evaluation of the Hirschsprung-associated enterocolitis (HAEC) score: a multicenter study of 116 children with Hirschsprung disease. J Pediatr Surg. 2018;53:708–17.

20.Gunadi, Sukarelawanto, A.V.R., Ritana, A. *et al.* Postoperative enterocolitis assessment using two different cut-off values in the HAEC score in Hirschsprung patients undergoing Duhamel and Soave pull-through. *BMC Pediatr* 20, 457 (2020). <https://doi.org/10.1186/s12887-020-02360-x>

We hope that the revisions from this review have greatly enhanced the manuscript and satisfied the reviewers and editor. We believe it is now ready for publication in "PLOS One."

On behalf of all authors,

Yours sincerely,

Gunadi, MD, PhD

Professor in Pediatric Surgery

Department of Surgery, Faculty of Medicine, Public Health and Nursing

Universitas Gadjah Mada/Dr. Sardjito Hospital

Yogyakarta 55281, Indonesia

Email: drgunadi@ugm.ac.id
