# Supplementary material for: Does surgical approach affect Hirschsprung-associated enterocolitis risk? A comparison between transanal Swenson-like and endorectal pull-throughs
Source: PLoS One. 2026 Jan 8;21(1):e0340813. doi: 10.1371/journal.pone.0340813 (PMC12782437; doi:10.1371/journal.pone.0340813)
Supplement: Attachment — Submitted filename: Dear Editor.docx [file pone.0340813.s005.docx]

Dear Editor-in-Chief,

I have reviewed the revised manuscript titled “Does surgical approach affect Hirschsprung-associated enterocolitis risk? A comparison between transanal Swenson-like and endorectal pull-throughs submitted for consideration to PLOS ONE. After careful evaluation of the revisions, I recommend acceptance with concerns.

The authors have comprehensively addressed all major concerns from my initial review and Reviewer 1 through specific textual changes, methodological clarifications, enhanced statistical reporting (e.g., corrected sample size calculation, OR/CI interpretations, post-hoc power), detailed HAEC scoring protocols, reorganized references eliminating temporal inconsistencies, and expanded limitations discussing retrospective biases. These improvements substantially enhance clarity, rigor, and scientific validity.

**Key Strengths of Revisions**

- HAEC scoring now includes Pastor/Frykman criteria details, dual cut-offs (4/10), record extraction methods, and reproducibility limitations.
- Discussion explicitly links predictor factors (e.g., albumin significant in TEPT only) to recent literature with pathophysiologic context.
- Sample size justified (minimum 48, all 70 cases included); subgroup limitations and type II error risks acknowledged.
- Additionally, I remain uncertain if this manuscript fully aligns with PLOS ONE's current scope emphasizing broad interdisciplinary impact, given its specialized pediatric surgery focus with limited generalizability (single-center, n=70). I suggest the editorial team give this further consideration for fit.

Thank you for the opportunity to re-review this strengthened work.

Sincerely,
Associate Professor
Department of Clinical Biochemistry
Faculty of Pharmacy
Isfahan University of Medical Sciences
[mohammadalipour@pharm.mui.ac.ir](mailto:mohammadalipour@pharm.mui.ac.ir)
(+98) 913-170-1793
WhatsApp: (+98) 939-817-0984PONE-D-25-45077_R1.pdf
